# Tuned by metals: the TET peptidase activity is controlled by 3 metal binding sites

**DOI:** 10.1038/srep20876

**Published:** 2016-02-08

**Authors:** Matteo Colombo, Eric Girard, Bruno Franzetti

**Affiliations:** 1 CNRS, IBS, F-38027 Grenoble, France; 2 CEA, DSV, IBS, F-38027 Grenoble, France; 3 Univ. Grenoble Alpes, Institut de Biologie Structurale (IBS), F-38027 Grenoble, France

## Abstract

TET aminopeptidases are dodecameric particles shared in the three life domains involved in various biological processes, from carbon source provider in archaea to eye-pressure regulation in humans. Each subunit contains a dinuclear metal site (M1 and M2) responsible for the enzyme catalytic activity. However, the role of each metal ion is still uncharacterized. Noteworthy, while mesophilic TETs are activated by Mn^2+^, hyperthermophilic TETs prefers Co^2+^. Here, by means of anomalous x-ray crystallography and enzyme kinetics measurements of the TET3 aminopeptidase from the hyperthermophilic organism *Pyrococcus furiosus* (PfTET3), we show that M2 hosts the catalytic activity of the enzyme, while M1 stabilizes the TET3 quaternary structure and controls the active site flexibility in a temperature dependent manner. A new third metal site (M3) was found in the substrate binding pocket, modulating the PfTET3 substrate preferences. These data show that TET activity is tuned by the molecular interplay among three metal sites.

Protein degradation occurs in the three life domains and it is involved in a wide variety of cellular processes such as protein quality control, amino acids pool renewal and as a carbon source provider^1^,^2^. Cytosolic protein degradation is carried out by different classes of proteases that are substrate specific or that are self-compartmentalized to shield the active site to avoid unspecific proteolysis^2^. Among self-compartmentalized proteases, a new class of dinuclear metalloaminopeptidases complex was recently discovered and named TET due to its tetrahedral shape^3^. TETs are dodecameric particles of 500 kDa and they were first discovered in archaea and then identified in bacteria and eukaryotes^3^,^4^,^5^,^6^,^7^. To date, three types of archaeal TETs have been identified and characterized based on their substrate specificity. TET1 is a glutamyl-aminopeptidase, TET2 has a broad specificity against neutral amino acids and TET3 shows a preference for positively charged residues such as lysine and arginine^3^,^8^,^9^,^10^. The physiological role of these dodecameric particles is not completely understood, although it has been found that TET homologues, DNPEP and PfM18AAP, are involved in blood pressure regulation in humans^5^,^11^,^12^ and participates in the hemoglobin degradation in *P. falciparum*^13^, respectively.

TETs dodecamers are characterized by a tetrahedral shape showing four openings on the facets. Each apex of the tetrahedron is formed by three subunits arranged along a three-fold axis. Each subunit has an active site that hosts two metal ions (M1 and M2) forming a dinuclear metal center. The classification of each metal site was derived from the nomenclature of Schechter and Berger^14^. M1 lays in the mouth of the PfTET3 active site, while M2 is adjacent to the specificity binding pocket (P1) that hosts the P1 side chain. P1 electrostatic potential is adapted to welcome positively (TET3), negatively (TET1) or neutral (TET2) charged residues^3^,^8^,^10^. TETs monomers are all formed by a proteolytic domain and a PDZ-like oligomerization domain. Each subunit interacts through two large interfaces. The dimerization interface is mediated by the PDZ-like domains that form the dimeric building block. The oligomerization interface is supported by both the proteolytic domain and the PDZ-like domain, driving the association of the dimeric building blocks into the dodecameric assembly^15^,^16^. It has been recently shown that TET dimers are present *in vivo*^17^ and that their activity against long peptides is considerably lower compared to the dodecameric particle^16^, suggesting a fine regulation of TET activity by controlling its oligomerization.

A key factor in TET oligomerization is the dinuclear metal site. In particular, when TET particle is treated with a high concentration of chelating agent (EDTA) associated with basic pH (pH 10) at room temperature, the dodecamer dissociates into dimers^18^. Indeed, the dinuclear metal site is close to the oligomeric interface and the depletion of metals may destabilize the H-bonds interactions between two adjacent subunits resulting in the deoligomerization of the TET particle^18^. The dinuclear metal center has not only a structural role in the TET particle, but it is also responsible for the TET catalytic activity. To date, there is no characterization of the role played by each metal in the active site of TET, although x-ray crystallography and extended x-ray absorption fine structure (EXAFS) studies have shown that the dinuclear metal center of TET can be occupied by Zn^2+^, Co^2+^ and Mn^2+^ ions which catalyses the aminopeptidase activity with different yields^4^,^8^,^9^,^10^. Interestingly, while the three thermophilic archaeal TETs require Co^2+^ to reach the optimal activity, their mesophilic counterparts *Streptococcus pneumoniae* PepA (SpPepA), DNPEP and PfM18AAP are activated by Co^2+^ and/or Mn^2+^. The molecular basis of Co^2+^ and/or Mn^2+^ activation has not been elucidated yet.

In this study, to get insights on the metal specificity of TET and in particular to address the specific role of each metal in the active site of the enzyme, the TET3 aminopeptidase from the hyperthermophilic organism *Pyrococcus furiosus* (PfTET3) was characterized by means of x-ray crystallography and UV-spectroscopy in the presence of Zn^2+^, Co^2+^ and Mn^2+^. In particular, crystallogenesis with controlled Co^2+^ concentration and subsequent characterization by x-ray anomalous experiment and enzymatic assays revealed that Co^2+^ replaces the M1 site and occupies a new third metal site (M3) in the specificity binding pocket. Importantly, the M2 site was not replaced by Co^2+^. Moreover, enzymatic assays performed on a wide range of temperature (20 °C–85 °C) have allowed determining the role of each metal ion. These results highlight a complex metal interplay at the base of the TET3 enzyme activity, establishing that the M1 site controls the flexibility of the active site, M2 hosts the activated water molecule and M3 modulates the substrate specificity for the enzyme. Finally, the molecular basis of Co^2+^ activation are depicted, showing that Co^2+^ increases the activity of PfTET3, by stabilizing the active site, only at high temperature.

## Results

### X-ray crystal structure of PfTET3 at 2.5 Ǻ resolution reveals that M2 is the catalytic metal

Previously, PfTET3 was assigned to M18 family and it was reported as a homotetramer based on gel filtration elution profile^19^. However, PfTET3 displays 90% of sequence identity with TET3 from *Pyrococcus horikoshii* (PhTET3), whose x-ray crystal structure was determined, showing a dodecameric assembly^10^. To unambiguously characterize PfTET3, we determined its crystal structure by SAD at 2.5 Å resolution. Purified PfTET3 was crystallized in HEPES 0.1M pH 7.7, NH_4_CH_3_COO 0.2 M, 2 mM CoCl_2_, MPD 44% and Gd-DO3A^20^ using hanging drop vapor diffusion method. Crystals appeared after 1 month and X-ray data collection was performed at ID29 beamline at the European Synchrotron Radiation Facility (ESRF-Grenoble). PfTET3_Gd_ crystal was collected at Gd LIII absorption edge. A strong anomalous signal relative to the Gd atoms extends up to 3.0 Å, allowing determining the Gd substructures. These latter were used to solve the phase problem relative to PfTET3. Data collection and refinement statistics are reported in Table 1.

**Figure 1:**
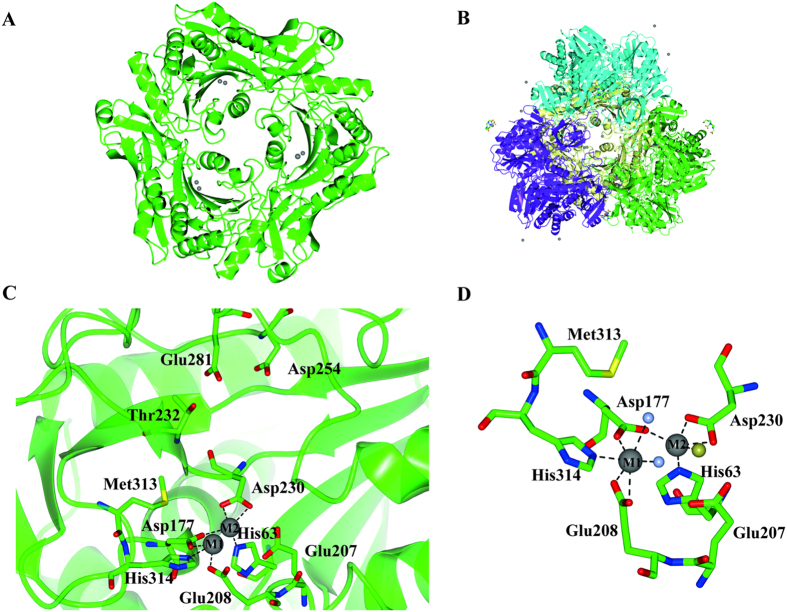
Structural features of PfTET3. **(A**) PfTET3 trimer in the asymmetric unit. The three monomers are almost identical with an r.m.s.d of 0.18 Å over the three chains. (**B)** PfTET3 dodecameric assembly obtained by applying the symmetry operators. Each trimer is highlighted by a different color (violet, green, yellow and cyan). Grey spheres represents metal ions. The sticks represent the DO3A bound to the Gd atoms. (**C)** Dinuclear active site and specificity binding pocket of one monomer. M1 site is coordinated by Asp177, Glu208 and His314 and M2 site is coordinated by His63, Asp177 and Asp230 (grey spheres). The identity of each metal ion is unknown. The specificity binding pocket is formed by Thr232, Asp254 and Glu281. (**D)** Zoom of the dinuclear metal center. Grey spheres are M1 and M2, gold sphere is Cl^−^ and ice blue spheres are water molecules. M1 has an octahedral geometry while M2 has a trigonal bipyramidal geometry.

The asymmetric unit contains three monomers forming one apex of the PfTET3 tetrahedron (Fig. 1A). By applying the crystal symmetry operators, PfTET3 dodecamer is reconstituted (Fig. 1B). Each monomer is formed by a proteolytic domain and a PDZ-like domain, known as TET dimerisation domain. Residues 120–128 are disordered and they were not modeled. The proteolytic domain comprises a central β-sheet surrounded by seven α-helices and accommodates the catalytic pocket. This latter contains two strong positive peaks of electron density that corresponds to the two metal ion sites. Each metal site was classified based on the nomenclature of Schechter and Berger^14^. M1 is coordinated by residues His314, Asp177, Glu208 and two water molecules, while M2 is coordinated by Asp177, Asp230, His63 and a chloride ion (Fig. 1C). In P1 pocket, Asp254 establishes H-bond with a water molecule. The purified PfTET3 contains two Zn^2+^ ions in the active site. However, since 2mM CoCl_2_ were present in the crystallization conditions, it can be inferred that a metal exchange occurred. Indeed, M1 displays an octahedral coordination, while M2 displays a trigonal bypiramidal coordination. By looking to the preferred coordination number of Co^2+^ and Zn^2+^, it appears that Co^2+^ adopts preferentially an octahedral geometry (coordination number of 6), while Zn^2+^ is often found in a tetrahedral coordination (coordination number 4)^21^. These data suggest that M1 is occupied by Co^2+^ while M2 is occupied by Zn^2+^. The two metals are separated by 3.3 Å in the three monomers present in the asymmetric unit (Fig. 1D). By comparison, in the PhTET3 structure the two Zn ions are separated by 3.4 Å (PDB 2WZN) while in SpPepA crystallographic structure, 12 monomers were found in the asymmetric unit (PDB 3KL9) and the distance between the two Zn ions varies between 3.1 Å - 3.5 Å, suggesting a dynamic positioning of the metal ions in the active site.

In the PfTET3 structure, M2 encompasses a Cl^−^ ion in his coordination sphere (2.8 Å). Indeed, it was reported that a carboxylate rich environment favors a hard acid behavior of the two metal ions in the active site resulting in the binding of halides in the order F^−^ >> Cl^−^> Br^−^> I^−^22. The catalytic mechanism of aminopeptidase involves a hydroxide ion, which is formed by the polarization of a water molecule bound to the two metal ions. Indeed, the Nδ and Nε of His63 are involved in the weak H-bonds network with the Oδ of Asp65 (3.1 Å) and the Oε of Glu207 (3.1 Å), respectively. Glu207 is the general base responsible for the deprotonation of the water molecule. It has been proposed that such an environment results in a decreasing of the acidity of the M2 metal^23^ allowing the polarization of the nucleophilic water molecule. It was previously reported that F^−^ ions act as a noncompetitive inhibitor by displacing the hydroxide ion from the metal ion^24^. Thus, the Cl^−^ ion can mimic the position of the hydroxide. In PfTET3_Gd_ structure, Cl^−^ ion is in the coordination sphere of M2, indicating that M2 is the catalytic metal.

Interestingly, M1 is coordinated by Glu208, which lays on a loop involved in PfTET3 oligomerization interface. In particular, Gln206 and Arg212 from one subunit, form H-bonds with the carbonyl carbon of Pro257 and Asn292 and Gln295 side chains of the adjacent subunits. This suggests that M1 may have a stabilizing role of PfTET3 oligomerization interface.

### M1 site is replaced by Co^2+^ in the PfTET3 active site

**Figure 2:**
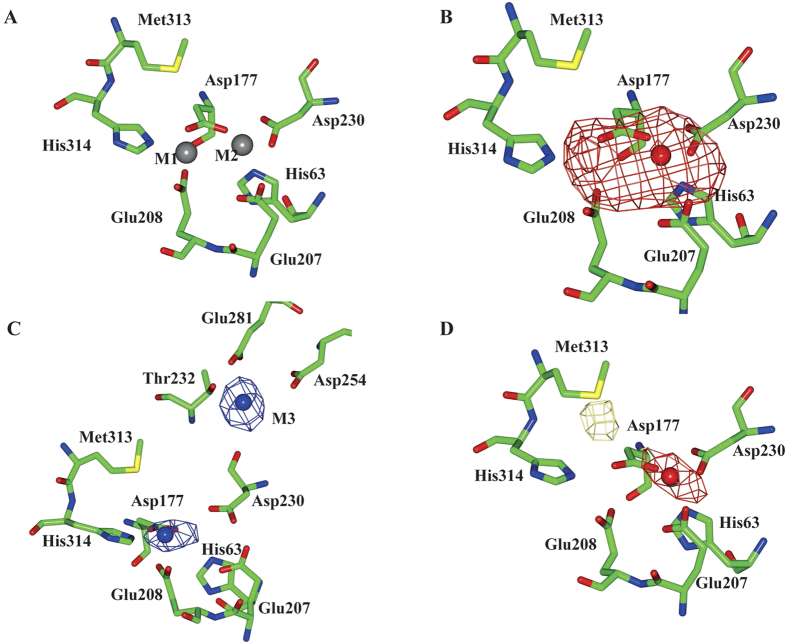
Determination of the metal sites replaced and/or occupied by Co^2+^ in the PfTET3 active site by anomalous x-ray scattering. Crystals of PfTET3 were grown in the presence of 2 Co^2+^ equivalents. Blue spheres are cobalt ions (M1 and M3), red sphere is zinc ion (M2). (**A**) Schematic representation of the PfTET3 active site. M1 is coordinated by Asp177, Glu208 and His314 and M2 is coordinated by His63, Asp177 and Asp230. Both sites are represented as grey spheres. (**B)** Anomalous Fourier map (red) of the PfTET3 catalytic site (contour 8σ) at Zn^2+^ absorption edge (both Zn^2+^and Co^2+^ display anomalous signal). M2 is highlighted by the anomalous Fourier map with an extra density for the M1 site. (**C)** Anomalous Fourier map (blue) of the PfTET3 catalytic site (contour 9σ) at Co^2+^ absorption edge (only Co^2+^ display anomalous signal). M1 and an additional metal site (M3) coordinated by Thr232, Asp254 and Glu281 are highlighted by the anomalous Fourier map. (**D**) Anomalous Fourier map (red and yellow) of the PfTET3 catalytic site (contour 4.5σ) at 40ev below Co^2+^ absorption edge. At this wavelength the anomalous signal from Co^2+^ is lower than that of Zn^2+^ or sulphur. Only the M2 site is highlighted by the anomalous Fourier map. The yellow map indicates the sulphur anomalous signal while the red map highlights the anomalous contribution from the zinc ion.

In the previous paragraph, PfTET3 *de novo* x-ray structure suggested that M2 is the catalytic metal. The three TETs from *Pyrococcus horikoshii* display enhanced enzymatic activity in the presence of Co^2+^, implying that M1 and/or M2 are exchanged^10^. To determine which metal site is exchanged by Co^2+^, anomalous x-ray scattering experiments were performed on PfTET3. Crystals of PfTET3 were grown at 20 °C in the presence of 2 Co^2+^ equivalents per monomer. For the sake of the clarity, hereafter, PfTET3 enzyme treated with Co^2+^ is reported as PfTET3_Co_ while the recombinant PfTET3 is named PfTET3_Zn_. PfTET3_Co_ crystals appear after 7–10 days, they were flash-frozen and x-ray data were collected on the same crystal at two different wavelengths corresponding to the Co^2+^ and Zn^2+^ absorption edges, respectively. The anomalous X-ray data for both Co^2+^ and Zn^2+^ were processed using XDS/Aimless/SHELXC/ANODE softwares. The positions of the anomalous scatterers (Co^2+^ and Zn^2+^) were determined using ANODE^25^. ANODE calculates an anomalous difference Fourier by applying a 90° phase shift to the protein phase that is calculated from a PDB model. We used the *de novo* PfTET3_Gd_ structure reported in this paper as a PDB model. At Zn^2+^ absorption edge, ANODE identifies four main peaks. Three peaks correspond to the M2 site of each monomer (A, B, C) and one corresponding to the M1 site in monomer C. Although in monomer A and B ANODE did not identify a clear peak for M1 site, residual electron density protruding from the M2 site is observed (Fig. 2B and Table 2). Indeed, at Zn K absorption edge, f” of Zn^2+^ is 3.9 e^−^ while f” of Co^2+^ is 2.4 e^−^. Conversely, at Co absorption edge, ANODE identifies three main peaks corresponding to M1 site for A, B, C, monomers. Interestingly, other three smaller peaks were also identified by ANODE in each monomer in the asymmetric unit, corresponding to a third metal site M3, coordinated by residues Glu281, Asp254 and Thr232 (Fig. 2C and Table 2). Interestingly, M3 coordinating residues are part of the PfTET3 specificity binding pocket. To confirm that Co^2+^ exchanges M1 site and occupy M3 site a further dataset was collected at 40ev below Co^2+^-edge (PfTET3_40eV_). At this energy, f” of Zn^2+^ and Co^2+^ are 0.77 e^−^ and 0.45 e^−^, respectively. Thus, it is expected that M2 site displays higher anomalous signal compared to M1. Furthermore, below Co^2+^-edge, sulphur displays significant anomalous signal (f” for S is 0.62 e^−^). Met313 is close to M1 site and it can be used as a probe to compare the anomalous signal. Indeed, ANODE found two small peaks corresponding to M2 site and Met313 in the active site (Fig. 2D and Table 2). These data show that Co^2+^ replaces the M1 site and occupies an additional M3 site, while M2 retains the Zn^2+^ ion recovered from the cell culture medium.

### M1 site in PfTET3 active site is implicated in the oligomeric interface stabilization

**Figure 3:**
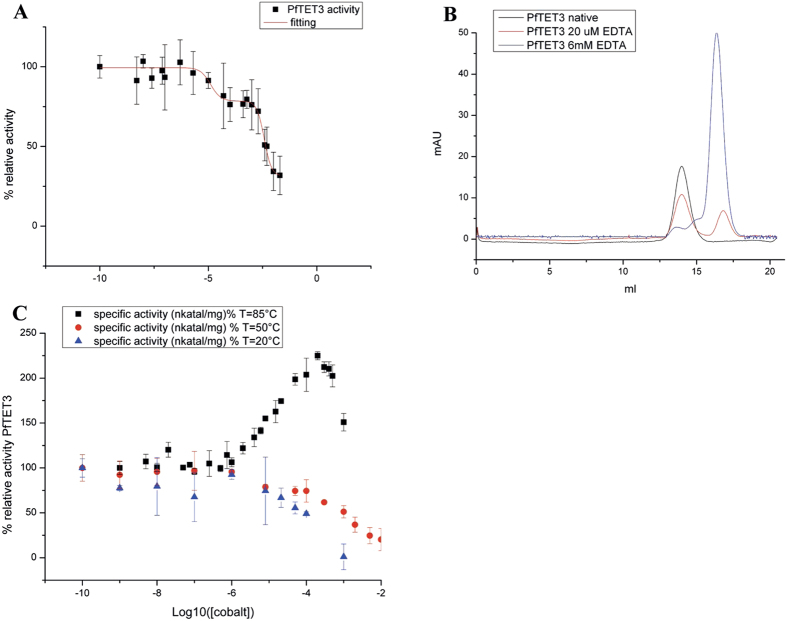
Enzymatic activity and oligomerization state of PfTET3 is modulated by M1. **(A**) EDTA titration of PfTET3 activity towards 5 mM Lys-pNa at T = 85 °C. Data are fitted by a bi-dose responsive curve from Origin 8.5 (red line). Error bars are standard deviations of three independent measurements. (**B)** Gel filtration (GF) profiles of PfTET3_Zn_ heated at 85 °C in the absence of EDTA (black line, dodecamer), with 15 μM EDTA (red line, 60% dodecamer and 40% monomer from GF profile)) and with 6 mM EDTA (blue line, monomer). (**C)** Co^2+^ titration of PfTET3 activity towards 5 mM Lys-pNa at T = 85 °C (black squares), T = 50 °C (red squares) and T = 20 °C (blue squares). Co^2+^ enhances the PfTET3 activity only at 85 °C.

In the presence of Co^2+^, the PfTET3_Zn_ M1 site is exchanged by Co^2+^, while M2 remains occupied by Zn^2+^. However, the respective role(s) of M1-M2 in the active site of PfTET3_Zn_ remain to be determined. It was previously reported for *Aeromonas proteolytica* aminopeptidase (AAP) and methionine aminopeptidase (MetAP)^23^,^26^ that 50–80% of the enzymatic activity can be obtained with only one metal in the active site. To evaluate the role of M1 and M2 in the PfTET3_Zn_ dinuclear metal site, an EDTA titration was performed at 85 °C on the PfTET3_Zn_ enzymatic activity using 5 mM Lys-pNa as substrate (Fig. 3A). The EDTA concentration used spans from 5 nM to 20 mM. Interestingly, the data points are fitted with a biphasic dose response curve (correlation coefficient = 0.97), indicating the presence of two distinct metal binding sites displaying different affinities. The first inflection point corresponds to an EC50_1_ = 13 μM, while EC50_2 _is 3.8 mM. After the first inflection point, PfTET3_Zn_ activity is still at 75%, while after the second inflection point it falls down to 30%. Based on the crystallographic studies reported above and the EDTA titration, the M1 site is the lower affinity site exchanged by Co^2+^, while the M2 is the high affinity site responsible for the catalytic activity of PfTET3_Zn_.

To evaluate the effect of the depletion of each metal ion on the PfTET3_Zn_ quaternary structure at its physiological temperature, 1 μM PfTET3_Zn_ was heated at 85 °C for 5 minutes in the absence of EDTA, in the presence of 15 μM EDTA (to selectively remove the M1 site) and in the presence of 6 mM EDTA (to remove both M1 and M2 sites), respectively. The samples were then loaded on a gel filtration column (Superose 6) and the UV profiles were analysed (Fig. 3B). PfTET3_Zn_ heated in the absence of EDTA is eluted as a dodecamer. The sample treated with 15 μM EDTA shows two peaks, corresponding to the dodecameric PfTET3_Zn_ and to the monomeric form of PfTET3_Zn_ based on column calibration. The dodecamer represents 60% of PfTET3_Zn_, while the monomer accounts for the remaining 40%. The sample treated with 6 mM EDTA shows a major peak, corresponding to the monomeric PfTET3_Zn_. These results show that the removal of the M1 site is sufficient to destabilize the quaternary structure of PfTET3_Zn_ that partially dissociates into monomers. The proximity of the M1 site to the oligomeric interface of PfTET3_Zn_ strongly suggests that the M1 site modulates the molecular interactions between adjacent subunits.

### **Co** ^**2+**^ **enhances PfTET3 activity only at Pyrococcus physiological temperature**

It has been shown that mesophilic TETs (DNPEP, SpPepA, PfM18) are activated by Co^2+^ and/or Mn^2+^, while Zn^2+^ being inhibitory^4^,^5^,^9^,^27^. Interestingly, while mesophilic TETs display enhanced activity in the presence of Co^2+^/Mn^2+^, hyperthermophilic TETs were activated only by Co^2+^. This observation prompted us to study the effect of temperature over the metal preference of PfTET3. PfTET3_Zn_ enzymatic activity was monitored by following the release of pNa from Lys-pNa substrate in the presence of two different concentrations (0.1 mM and 1 mM) of Co^2+^, Mn^2+^ and Zn^2+^ and at three different temperatures (T = 20 °C, 50 °C, 85 °C). At 20 °C and 50 °C, the addition of each type of metal ions inhibited the enzymatic activity of PfTET3_Zn_. However, Mn^2+^ resulted the less inhibitory metal at T < 50 °C (Table 3). At 85 °C, PfTET3_Zn_ activity is enhanced by Co^2+^ addition while being strongly inhibited by Mn^2+^ and Zn^2+^. These results indicate a specific effect of Co^2+^ on PfTET3 enzyme; activator at physiological temperature and inhibitor at T < 50 °C. Furthermore, Table 3 shows that at 85 °C and 1 mM Co^2+^, PfTET3_Zn_ activity is less enhanced, revealing that Co^2+^ concentration is also important to regulate the enzyme activity. These results prompted us to perform a Co^2+^ titration of PfTET3_Zn_ activity at 20 °C, 50 °C and 85 °C. The results are shown in Fig. 3C. The plots confirmed the activator role of Co^2+^ only at 85 °C, while at 20 °C and 50 °C Co^2+^ inhibits PfTET3 enzymatic activity. The data points at 85 °C describe a growing sigmoidal curve up to 230% of PfTET3_Zn_ activity, while representing a decreasing curve from 230% to 150% of the PfTET3_Zn_ activity. The data points at 85 °C were fitted with two dose-response functions, one from 0% up to 230% of PfTET3_Zn_ activity and the second from 230% to 150% of PfTET3_Zn_ activity. These two dose-response functions allowed determining two EC50 values, EC50_1_ of 7.4 μM and EC50_2_ of 550 μM. EC50_1_ represents the Co^2+^ concentration value at which half of the maximal enzyme activity is reached. Interestingly EC50_1_ value of the Co^2+^ titration (7.4 μM) is similar to the EC50_1_ found in the EDTA titration (13 μM). Based on the structural studies reported above as well as on the EC50_1_ values of the Co^2+^/EDTA titration, the Co^2+^ mediated enhancement of the PfTET3 activity is dependent from the M1 site. Furthermore, it suggests that the maximum activity of PfTET3 is reached by a hybrid dinuclear metal site, with M1 and M2 filled with Co^2+^ and Zn^2+^, respectively.

### **Co** ^**2+**^ **at the M1 site modulates the PfTET3 active site flexibility**

To shed light on the role played by Co^2+^, the kinetic parameters of PfTET3 were measured at 85 °C in the presence/absence of 300 μM Co^2+^ and using Lys-pNA as substrate. Results are reported in Table 4. Interestingly, in the presence of Co^2+^, there is a strong reduction of Km compared to the recombinant PfTET3_Zn_ (0.6 mM vs 7 mM). Conversely, kcat is similar with or without Co^2+^. To quantify the thermodynamic contribution of Co^2+^ on PfTET3 activity, kcat/Km ratio of each PfTET3 variant were inserted in the Haldane equation^28^, that allow to determine the difference in transition state free energies (ΔΔG_ES_^†^) between PfTET3_Co_/PfTET3_Zn_. ΔΔG_ES_^†^ resulted of −6.7 kJ/mol. These results demonstrate that Co^2+^ allows the stabilization of the transition state at the physiological temperature of the PfTET3 enzyme.

**Figure 4:**
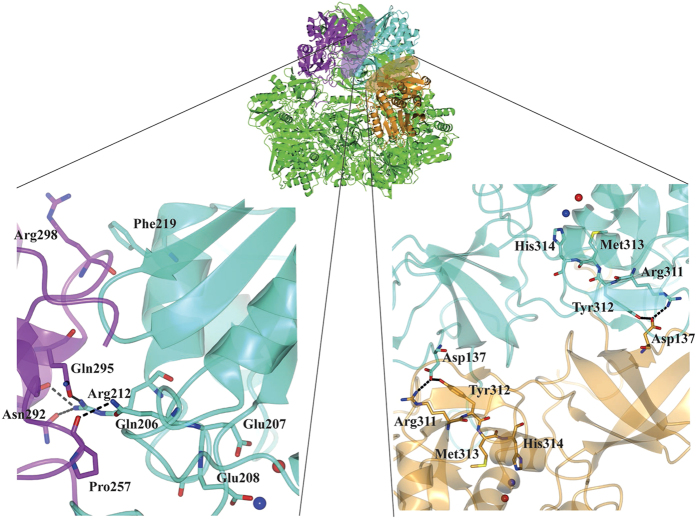
M1 site affects the stability of the PfTET3 oligomeric and dimeric interfaces. On the top, the purple ellipse in the dodecamer highlights the oligomeric interface (formed by subunits purple and cyan) and the golden ellipse highlights the dimeric interface (formed by subunits gold and cyan). Left panel, inset of PfTET3 oligomeric interface (purple and cyan subunits). M1 and M2 sites are represented as a blue and a red sphere, respectively. The M1 coordinating residue, Glu208, lay on a loop that hosts Gln206, Arg212 (cyan). These residues are involved in H-bonding (represented by dashed lines) with Pro257, Asn292 and Gln295 of the adjacent subunit (purple). Additionally, Phe219 establishes π-stacking interactions with Arg298 of the adjacent subunit. Noteworthy, such interface is repeated 12 times in the dodecamer. Right panel, inset of PfTET3 dimeric interface (golden and cyan subunits). His314 coordinating the M1 site is close to the PfTET3 dimeric interface. In particular, Arg311 and Tyr312 are involved in salt bridges (represented with dashed lines) with Asp137 of the adjacent subunit. Noteworthy, each dimeric interface contain two copies of such interactions due to the 2-fold symmetry of the dimeric interface.

**Figure 5:**
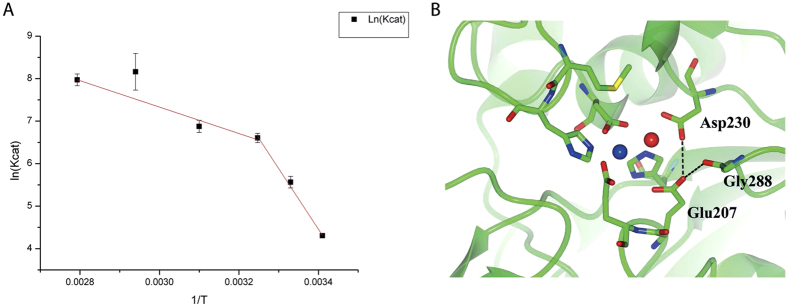
Temperature dependence of PfTET3 activity. **(A**) Arrhenius plot of PfTET3_Zn_ between 20 °C–85 °C. The breakpoint is at 35 °C, suggesting a reduced flexibility of PfTET3_Zn_ in the active site and at the interfaces. The non-linear profile indicates cold inactivation by rigidifying the oligomeric interface. (**B)** PfTET3 active site. Blue and red spheres stay for Co^2+^ and Zn^2+^, respectively. For the sake of the clarity, only residues involved in salt bridges with Glu207 are labeled. The dashed lines are the electrostatic interactions between Glu207 and Gly288/Asp230. Such interactions increase the pKa of Glu207 and decrease the enzymatic activity of PfTET3 at 20 °C.

To get more insights on this phenomena, the kinetic parameters of PfTET3_Zn_ were measured at five additional temperatures (20 °C, 27.5 °C, 35 °C, 50 °C, 67.5 °C) using Lys-pNA as substrate and an Arrhenius plot was built. It has been reported that thermophilic enzymes may display cold inactivation and they display a non-linear Arrhenius plot^29^. Recently, a non-linear Arrhenius plot for the tetrameric thermophilic enzyme, alcohol dehydrogenase was published^30^. Based on site-directed mutagenesis at the active site of the alcohol dehydrogenase and the thermodynamic parameters determined (ΔH^‡^, TΔS^‡^ and the Arrhenius pre-factor A_obs_) , the authors proposed a direct link between temperature and protein-protein interfaces flexibility, suggesting that at low temperature, the interfaces becomes steeper resulting in the impairment of the alcohol dehydrogenase activity^30^,^31^. Interestingly, our data reported above have shown that the M1 site exchanged by Co^2+^, determines a stabilization of the transition state of the PfTET3 enzyme. Furthermore, the M1 site is close to the dimeric and oligomeric interfaces of PfTET3 (Fig. 4). These observations lead us to build an Arrhenius plot for PfTET3_Zn_ by plotting the kcat vs 1/T. The plot resulted non-linear, with a break at 35 °C (Fig. 5A). The data points were fitted by two straight lines, the first covering the points 20 °C < T < 35 °C and the second covering the points 35 °C < T < 85 °C.

Both straight lines are described by equations of the type ln(kcat) = −Ea/R*1/T + lnA_obs_, where Ea is the energy of activation, R is the gas constant and A_obs_ is the Arrhenius pre-factor. From the slope of both straight lines Ea has been calculated. Interestingly, in the range 20 °C< T < 35 °C, Ea is high (118 kJ/mol) while in the range 35 °C < T < 85 °C Ea value decreases to 25.8 kJ/mol. The values of the enthalpy of activation (ΔH^‡^), the entropy of activation (TΔS^‡^) and of the A_obs_ are listed in Table 5. It is noteworthy that the A_obs_ below 35 °C adopts a value of 10^22^ while above 35 °C A_obs_ is 10^7^. The high value of A_obs_ below 35 °C indicates an unusual temperature dependence compared to the A_obs_≈ 10^13^ observed for a number of chemical reactions modeled by transition state theory^32^. Furthermore, in the range 20 °C–35 °C, ΔH^‡^ adopts a high value (116 KJ/mol). Noteworthy, this high energy barrier is compensated by a high positive TΔS^‡^, thus implying an increased flexibility of the PfTET3 active site. Indeed, it has been reported that flexibility may lower the energy barrier of the reaction by increasing the number of enzyme conformational substates, resulting in a higher probability for the reaction to occur^33^,^34^.

Conversely, in the range 35 °C–85 °C, ΔH^‡^ dropped to 23 KJ/mol, while TΔS^‡^ became negative (−41 KJ/mol) reflecting an increased rigidity of the active site.

However, a change in rate-limiting step may be at the base of the non-linear Arrhenius plot. In the aminopeptidase enzymatic reaction, the rate limiting step is governed by the general base that deprotonates the water molecule between the two metal ions. In the case of PfTET3, Glu207 is the general base. To evaluate the effect of temperature on the rate limiting step of the enzymatic reaction catalysed by PfTET3, the pKa value of Glu207 was determined at 20 °C and 85 °C in the presence/absence of 300μM Co^2+^ in the pH range 4–8.7. By plotting the pH on the x-axis and the log(kcat) on the y-axis, a graph was built (data not shown). The data points were fitted with a sigmoidal function, allowing the calculation of the inflection point corresponding to the Glu207 pKa. Interestingly, at 20 °C Glu207 pKa is 5.9 and 5.6 for PfTET3_Zn_ and PfTET3_Co_, respectively (Table 5). At 85 °C Glu207 pKa decreases down to 4.7 in PfTET3_Zn_ and to 4.4 in PfTET3_Co_ (Table 6). This means that at low temperature, the PfTET3 enzymatic reaction is chemically unfavored in both variants. The acidity of Glu207 is affected by the amino acids in its surroundings. Interestingly, in the PfTET3_Gd_ structure here reported, Glu207 establishes repulsive interactions with the carbonyl carbon of Gly288 (3.3 Å) as well as with the carboxylic side chain of Asp230 (2.7 Å) (Fig. 5B). These interactions diminishes the acidity of Glu207 resulting in an elevated pKa at 20 °C. At 85 °C, the pKa value of Glu207 suggests that the distances with residues Gly288 and Asp230 are increased, resulting in a more efficient enzymatic reaction. Furthermore, the pKa value changes only slightly in the presence of Co^2+^, suggesting that the observed Co^2+^ effect on PfTET3 activity is not directly linked to the pKa variation of Glu207. Indeed, the kcat values for PfTET3_Zn_ and PfTET3_Co_ are very similar, while their Km differs of a factor of 10. These results support the idea that M1 site is devoted to the regulation of the flexibility of the PfTET3 active site to optimize the enzyme activity at the physiological temperature.

### The M3 site allows the degradation of negatively charged residues by PfTET3

**Figure 6:**
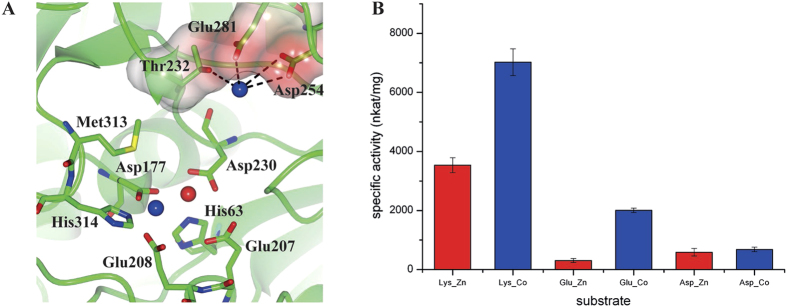
The M3 site broaden the PfTET3 substrate preferences. **(A**) P1 pocket of PfTET3 in electrostatic surface representation. Blue spheres are Co^2+^ ions, red sphere is a Zn^2+^ ion. For the sake of the clarity, only the residues interacting with the M3 site are highlighted. The presence of M3 modify the electrostatic properties of P1 allowing the degradation of glutamate. (**B)** Substrate specificity of PfTET3 is expanded towards glutamate in the presence of Co^2+^ (blue rectangles) at 85 °C. In the absence of Co^2+^ (red rectangles) PfTET3 is almost inactive. Error bars are standard deviations of three independent measurements.

The specificity binding pocket (P1) of PfTET3 is devoted to the substrate side-chain accommodation and it is formed by Thr232, Asp254, Glu281, Thr285 and Thr287. This pocket is negatively charged and it allows the degradation of positively charged residues such as lysine and arginine. The evidence of the Co^2+^ bound in P1 pocket (M3) may change the substrate preferences of PfTET3_Co_ (Fig. 6A). In particular, the presence of the cation could allow the coordination of negatively charged side chains and their subsequent degradation. To examine this possibility, PfTET3 activity was tested at 85 °C by using 5 mM Glu-pNa and Asp-pNa as substrates, in the presence/absence of 300 μM Co^2+^. Results are reported in Fig. 5B, showing that only PfTET3_Co_ degrades Glu-pNa (Fig. 6B). The activity of PfTET3_Co_ on Glu-pNa represents nearly 30% of the PfTET3_Co_ activity on Lys-pNa. Interestingly, aspartate is not degraded efficiently. The distance between the M1-M2 sites and M3 is 9.5Å. This length can be covered by a long side-chain, such as lysine, arginine and glutamate. Conversely, aspartate side chain is too short and thus cannot be accommodated efficiently in P1. These results suggest that the M3 site allows extending the type of substrates that can be degraded by PfTET3_Co_.

## Discussion

One third of all proteins contain metal ions that are devoted to catalysis and structural stability 35. Among these, metallopeptidases cover a large portion of the metalloproteins. Metallopeptidases are present in a wide variety of oligomeric states, from monomeric (methionine aminopeptidase, *Aeromonas proteolytica* Aminopeptidase)^36^,^37^ to higher oligomeric states (leucine aminopeptidase, tricorn peptidase complex and TET)^3^,^38^,^39^. They all contain one or two metals in the active site that support the catalytic activity of the peptidase. Moreover, it has been shown that different metals in the active site of some of these proteases lead to dramatic variations of the enzyme kinetic parameters^40^. While monomeric peptidases have been extensively studied to determine the role of each metal ion in the active site^23^,^24^,^41^, there is a lack of information regarding the role of each metal ion in oligomeric peptidases. Here, by means of anomalous X-ray crystallography coupled to enzyme kinetics, we have characterized the respective role of each metal ion in the hyperthermophilic PfTET3 aminopeptidase and determined the molecular basis of Co^2+^ activation.

In the x-ray structure of PfTET3_Gd_ a Cl^−^ ion is coordinated by the M2 site (Fig. 1D). Similarly, it was reported for the monomeric *Streptomyces* dinuclear aminopeptidase that F^−^ ion replaces the hydroxide ion on the catalytic metal, resulting in the enzyme activity inhibition^24^. Thus, the position of the Cl^−^ ion in PfTET3_Gd_ structure strongly suggest that M2 is the catalytic metal hosting the nucleophilic hydroxide. This observation is confirmed by the anomalous x-ray experiments performed on PfTET3 crystals grown in the presence of 2 Co^2+^ equivalents. In this case, the Zn^2+^ present in the M2 site is not replaced by Co^2+^ (Fig. 2). Indeed, the kinetics measurements show that the higher catalytic efficiency kcat/Km of PfTET3_Co_ compared to the kcat/Km of PfTET3_Zn_ is due to a lower Km more than to a higher kcat Table 4).

Conversely, the anomalous x-ray experiments on PfTET3 crystals grown in the presence of Co^2+^ have shown that the M1 site is replaced by Co^2+^. Interestingly, the M1 site is coordinated by Asp177, Glu208 and His314. Noteworthy, Glu208 and His314 residues lay on loops implicated in H-bonds between adjacent subunits, mediating the oligomeric and the dimeric interfaces, respectively (Fig. 4C,D). Indeed, the removal of the M1 site by EDTA is sufficient to partially dissociate PfTET3 into monomers (Fig. 3A,B). Furthermore, next to Glu208, there is Glu207 which is the general base that deprotonates the water molecule which in turn performs the nucleophilic attack on the C-N bond of the incoming substrate. Our data revealed that the pKa of Glu207 varies with temperature, adopting optimal values for catalysis at the physiological temperature of the PfTET3 enzyme (Table 6). Moreover, the pKa of Glu207 varied only slightly by Co^2+^ addition. This is in agreement with the slight variation of kcat values observed between PfTET3_Co_ and PfTET3_Zn_ variants (Table 4). Furthermore, it is noteworthy that the ratio kcat/Km of PfTET3_Co_ is ten times higher than that of PfTET3_Zn_. The kcat/Km parameter relates not only on the enzyme-substrate complex (as kcat) but also on the free enzyme^42^. This means that PfTET3_Co_ active site is more stable and catalytically active at 85 °C than the PfTET3_Zn_ counterpart. Thus, the M1 site shows a critical role in maintaining the quaternary structure of TET and modulating the flexibility of the enzyme active site. It is noteworthy that the dodecameric PfTET3 particle has twelve M1 sites that are involved in the stabilization of the overall macromolecular architecture. This is demonstrated by the non-linear Arrhenius plot here reported (Fig. 4A). It has been suggested that such a plot may arise for thermophilic enzymes and it represents a reversible distribution of the protein conformations displaying inactive states at low temperature^30^,^31^. Although the observed break in the Arrhenius plot here reported may be due to the higher pKa observed for Glu207 at 20 °C compared to 85 °C, it is noteworthy that at both temperatures the addition of Co^2+^ has a slight effect on the Glu207 pKa value, while it has a strong impact on the catalytic efficiency (kcat/Km) of PfTET3 at its physiological temperature. Furthermore, the addition of Co^2+^ at low temperature inhibits PfTET3 activity at a similar concentration at which Co^2+^ enhances PfTET3 activity at 85 °C (Fig. 3C). This means that the replacement of the M1 site by Co^2+^ at 20 °C < T < 50 °C may impair the equilibrium between stability and flexibility at the active site and at the interfaces of PfTET3 dodecamer. At low temperature, the hyperthermophilic PfTET3 enzyme has a high enthalpic energy barrier (116 KJ/mol) to overcome. This is attenuated by the high positive value of TΔS‡ (53 KJ/mol) in the range 20 °C–35 °C. However, the addition of Co^2+^ may decrease the flexibility of the active site in the range 20 °C–35 °C, that is needed to counterbalance the high ΔH^‡^ in this temperature range. Conversely, in the range 35 °C–85 °C, ΔH^‡^ is lower (23KJ/mol) and TΔS‡ adopts a negative value, reflecting a higher conformational rigidity. In this case, the addition of Co^2+^ should favor the enzymatic reaction. Indeed, PfTET3 enzymatic activity is enhanced by Co^2+^ at 85 °C (Table 3 and Fig. 3C). The observed effects of Co^2+^ are physiologically relevant, since recent studies on the metallome of *P. furiosus* revealed a high intracellular content of Co^2+^, compared to *E. coli*^43^.

Because our thermodynamic data showed that PfTET3 transition state is stabilized by Co^2+^ at 85  °C and our structural data identified the M1 site as the metal site exchanged by Co^2+^, we propose that the M1 site has a stabilization role on the PfTET3 active site. These data indicate that PfTET3 shows the optimal activity with an hybrid dinuclear metal site, with M2 and M1 occupied by Zn^2+^ and Co^2+^, respectively. Interestingly, in monomeric aminopeptidases such as AAP and MetAP, the M1 and M2 sites appear reversed compared to PfTET3 and the enzymes do not need two metals in their active site to be active^40^. Conversely, the oligomeric leucyl aminopeptidase strictly needs the presence of two metals for its activity.

The x-ray data collected at Co^2+^ absorption edge highlighted two metal sites occupied by Co^2+^, the M1 site and a new M3 site. The M3 site is coordinated by residues of the P1 pocket (Fig. 5A). Interestingly, an M3 site has been observed in alkaline phosphatase^44^,^45^. In this enzyme, M3 may be occupied by Zn^2+^, Co^2+^ or Mg^2+^and is coordinated by Glu, Asp and Thr side chains in addition to three water molecules. In the structure of PfTET3_Co_ here presented, M3 is coordinated by the same protein ligands. Moreover, a M3 site has been observed in methionine aminopeptidases where it is coordinated by two His residues, three water molecules and by a Pro-Leu inhibitor molecule^26^,^36^,^46^. Furthermore, in endonucleases, a functional equivalent metal binding site has been proposed to act as stabilizing the negative charge of the phosphate transition state, by coordinating a water molecule that protonate the 3’ oxygen of the leaving group^47^,^48^. In this study we report for the first time a new function for the M3 site. Indeed, by modifying the electrostatic properties of the P1 pocket of the dodecameric aminopeptidase PfTET3, M3 allows the degradation of a negatively charged substrate (glutamate). Furthermore, it is noteworthy that M3 does not interfere with positively charged substrates (Fig. 5B). Indeed, at the same Co^2+^ concentration by which PfTET3 degrades glutamate, it also processes lysine. This observation suggests that M3 may also be responsible for the stabilization of the negatively charged transition state, as occurs in endonucleases and alkaline phosphatase. The presence of M3 has important biological implications by considering the recently characterized hetero dodecameric assembly in *Pyrococcus horikoshii* formed by six TET2 subunits and six TET3 subunits, identified *in vivo* and characterized *in vitro*^15^,^17^. This TET2_TET3 hetero dodecamer resulted more efficient in degrading complex peptides than the two separated homo-dodecamers of TET2 and TET3. The M3 site here presented represents a further upgrade in peptide processing, allowing the TET2_TET3 hetero dodecamer to be active on a wide variety of peptides.

In summary, this study allowed the characterization of the role of each metal site in the TET enzymes. The three metals are key players for the effective catalytic activity of TET as well as for the flexibility modulation of the active site. In particular, Co^2+^ may ensure the optimal geometry of the PfTET3 active site at high temperature. To our knowledge, it is the first time that such molecular interplay among three metal ions is observed for an oligomeric aminopeptidase. Furthermore, these data depict a connection between the organism living temperature and the metal selection in the TET active site. We speculate that the choice of metal in the active site of TET may be determined by the organism living temperature. It is noteworthy that the optimal concentration of Co^2+^ (300 μM) enhancing PfTET3 activity *in vitro* reported in this paper is compatible with the range of *in vivo* Co^2+^ concentration (from 85μM to 9 mM) found in the surface soil of Volcano island in Sicily, where *Pyrococcus furiosus* was first isolated^49^. Intriguingly, mesophilic TET aminopeptidases such as DNPEP and PfM18 are preferentially activated by Mn^2+^^4^,^7^, while Co^2+^ promotes their activity to a lower extent. It could be of interest to study the molecular basis that lead the mesophilic TET to select Mn^2+^ instead of Co^2+^.

## Materials and Methods

### Production and purification of PfTET3

PfTET3 was cloned in vector pET-41c by RoBioMol (RoBioMol platform at the IBS (CEA/CNRS/UJF), Grenoble). Expression and purification were carried out in the same way as for PhTET3^10^. The final step is a gel filtration in buffer Tris 20 mM pH 7.5, NaCl 300 mM. PfTET3 was concentrated in Amicon Ultra 100 kDa at a final concentration of 8 mg/ml for crystallization.

### Crystallization of PfTET3

Initial crystal hits were obtained by using the HTX lab platform at the EMBL, Grenoble^50^. The crystal conditions were optimized with hanging drop vapour diffusion method. The final crystal conditions were HEPES 0.1 M pH 7.7, MPD 44%, NH_4_CH_3_COO 0.2 M. Then, three types of PfTET3 crystals were prepared: PfTET3_Gd_ to determine the PfTET3 x-ray structure *de novo*, PfTET3_Co_ to evaluate stoichiometric metal exchange in the active site and PfTET3_Zn_ to show the presence of two metal sites after purification. PfTET3_Gd_ crystals were grown in HEPES 0.1 M pH 7.7, MPD 44%, NH_4_CH_3_COO 0.2 M and CoCl_2_ 2 mM and the drops were produced by adding 1.5 μl PfTET3 8 mg/ml + 1.5 μl reservoir + 1.5 μl 300 mM Gd-DO3A. Gd-DO3A is a lanthanide complex designed to obtain high-phasing power heavy atom derivatives. In this complex, a Gd^3+^ ion is coordinated by a tetraazacyclododecane macrocycle and two water molecules. By interacting with a carboxylic group from a macromolecule (aspartate or glutamate), the lanthanide complex loses one water molecule and establishes a complex with the macromolecule. CoCl_2_ was added because ameliorates the quality of the crystals. PfTET3_Co_ crystals were grown in HEPES 0.1 M pH 7.7, MPD 44%, NH_4_CH_3_COO 0.2 M and CoCl_2_ 0.4 mM and the drops contained 1.5 μl PfTET3 8mg/ml + 1.5 μl reservoir.

### Crystal structure determination of PfTET3_Gd_

PfTET3_Gd_ crystals were cryo-cooled with liquid nitrogen in mother liquor. X-ray diffraction intensities were collected on ID29 at the European synchrotron radiation facility, in Grenoble (ESRF) at Gd LIII wavelength, after energy scan. Data were processed with XDS^51^ and Aimless^52^. Phase shift (α) estimation of Gd atoms was made using SHELXC^53^. The resolution was initially cut at 3.0 Å based on the strength of the anomalous signal estimated by d”/sig (d”/sig as implemented in shelx^53^ software represents the anomalous differences divided by their estimated standard deviation). These initial substructures were input in PHASER, using SAD setup^54^ extending resolution up to 2.5 Å. Nine additional substructures were found and initial PfTET3 phases were determined by SHELXE^53^. Three cycles of density modification were performed by cparrot^55^, obtaining an initial FOM = 0.73. Initial model building was performed by BUCCANEER^56^ and the model was completed manually using COOT^57^. Model refinement was performed by PHENIX^58^. To calculate the R_free_, 5% of the reflections were excluded throughout the refinement process. Data collection statistics are listed in Table 1.

### Determination of the heavy atoms sites with ANODE of PfTET3_Zn_, PfTET3_Co_ and PfTET3_40eV_

PfTET3_Co_ crystals were cryo-cooled with liquid nitrogen in mother liquor. X-ray diffraction intensities were collected on PROXIMA II beamline at the french synchrotron SOLEIL, in Paris. Data were collected at Co^2+^-edge and Zn^2+^-edge. The absorption edges were evaluated with a fluorescence spectrum. The intensities were processed with XDS^51^ and Aimless^52^. Data reduction revealed the same tetragonal space group (98) observed in *de novo* PfTET3_Gd_ pdb model. Matthews’s coefficient proposes three monomers (A, B and C) in the ASU at both Zn^2+^edge and Co^2+^ edge. In both cases molecular replacement solutions confirmed Matthews’s coefficient estimation. Then, phase shift (α) estimation of cobalt and zinc atoms were successively calculated by SHELXC^53^ cutting the resolution at 3.35 Å and 3.25 Å for Co^2+^ and Zn^2+^ datasets respectively. The files produced by SHELXC were then input in ANODE together with the PDB model of PfTET3_Gd_ depleted of metal ions. The number of sites found by ANODE for both datasets is listed in Table 2. Data collection statistics are listed in Table 1. Images were prepared using CCP4mg software^59^.

### PfTET3_Zn_ titration by Co^2+^ or EDTA

10 nM PfTET3_Zn_ (relative to monomer) was titrated with CoCl_2_. The enzymatic activity was measured using 5 mM Lys-pNa as substrate at T = 20 °C, 50 °C and 85 °C in the buffer 0.1 M HEPES pH 7.2, KCl 316 mM and 0 mM −5 mM CoCl_2_. At T = 20 °C, 100 nM PfTET3_Zn_ was used due to the lower activity of PfTET3_Zn_ enzyme at this temperature. The same procedure was adopted for the EDTA titration, but the measurements were executed only at 85 °C and using 0 mM- 20 mM EDTA. All buffer solutions were pre-warmed and the pH is referred to the working temperature. Measurements were performed in triplicate. The reported specific activities were calculated as described above. 100% of the activity corresponds to the activity of the PfTET3_Zn_ enzyme.

### **PfTET3**_**Zn**_ **oligomerization in the presence of EDTA**

Three reaction tubes of 500 μl of HEPES pH 7.2 (at working temperature), KCl 316 mM containing 1 μM PfTET3_Zn_ alone, 1 μM PfTET3_Zn_ + 15 μM EDTA and 1 μM PfTET3_Zn_ + 6 mM EDTA respectively, were heated at 85 °C for 5 minutes. The tubes were subsequently cooled at room temperature and loaded on a Superose 6 column, preequilibrated with the same buffer used for the activity assay, without EDTA.

### Kinetics of PfTET3 enzymatic activity on chromogenic substrates

PfTET3_Zn_ and PfTET3_Co_ enzymatic activities were determined by monitoring the release of pNA at λ = 405 nm from different substrates (lysine, glutamate, aspartate) at T = 20 °C, 27 °C, 35 °C, 50 °C, 67.5 °C and 85 °C. The reaction mixture contained 10 nM PfTET3 (relative to monomer), 0.1 M HEPES pH 7.2, KCl 316 mM, 5 mM substrate at a final volume of 100 μl. pH is referred to the working temperature. All buffer solutions were pre-warmed. Only for the measurements at 20 °C, PfTET3 was at 100nM final concentration. Measurements were performed in triplicate by using a multi-cuvette JASCO V-630 UV-visible spectrophotometer. Temperature was controlled by a Peltier system. The enzymatic reaction was monitored along 10 minutes at 20 °C and for 5 minutes at higher temperatures. To calculate the specific activity, the linear slope (A/t) of the enzymatic reaction corresponding to the steady-state of the reaction was determined by using Spectra analysis software provided by Jasco. The slope was converted to V_0_ (M/s) using the pNA extinction coefficient (ε) (10000 M^−1^cm^−1^). The specific activity was then calculated by dividing V_0_ per PfTET3 added volume and PfTET3 mg/ml concentration. Plots were drawn using Origin software version 8.5. The kinetic parameters (Km, kcat and kcat/Km) were obtained by plotting V_0_ as a function of substrate concentration (0.2 mM – 20 mM). Data points were fitted by the Hill equation provided by Origin 8.5 (y = V_max_ * (x^n^/Km+x^n^)) and fixing term n = 1 to get the Michaelis-Menten equation.

### Effect of metal cations on PfTET3_Zn_ activity as function of temperature

Purified PfTET3_Zn_ enzyme activity was measured using 5 mM Lys-pNa as substrate at T = 20 °C, 50 °C and 85 °C in the buffer 0.1 M HEPES pH 7.2, KCl 316 mM and individually supplied with 0.1 mM / 1mM of CoCl_2_, MnCl_2_ and ZnCl_2_. All buffer solutions were pre-warmed and the pH is referred to the working temperature. Measurements were performed in triplicate. The reported specific activities were calculated as described above.

## Additional Information

**Data availability**: The coordinates of PfTET3Gd model and their experimental intensities are deposited in the PDB database (PDB 4X8I). The original diffraction images collected for PfTET3Co, PfTET3Zn and PfTET340eV were deposited in SBGrid60.

**How to cite this article**: Colombo, M. *et al.* Tuned by metals: the TET peptidase activity is controlled by 3 metal binding sites. *Sci. Rep.* **6**, 20876; doi: 10.1038/srep20876 (2016).

## Figures and Tables

**Table 1:** Data collection and refinement statistics for PfTET3_Gd_, PfTET3_Zn_, PfTET3_Co_, PfTET3_40eV._

Structure	PfTET3_Gd_ Gd peak	PfTET3_Co_ Co peak	PfTET3_Zn_ Zn peak	PfTET3_40eV_ low Co remote peak
Beam line	ID29 (ESRF)	Proxima II (SOLEIL)	Proxima II (SOLEIL)	Proxima II (SOLEIL)
Space Group	Tetragonal I4_1_22	Tetragonal I4_1_22	Tetragonal I4_1_22	Tetragonal I4_1_22
Unit Cell Constants (Å)	a = 203.91 b = 203.91 c = 112.44	a = 201.7 b = 201.7 c = 113.2	a = 201.7 b = 201.7 c = 113.4	a = 202.6 b = 202.6 c = 113.6
Resolution (Å)	48.06 − 2.50 (2.60 − 2.50)	49.37 − 3.35 (3.62 − 3.35)	49.42 − 3.25 (3.56 − 3.25)	49.53 − 3.30 (4.37 − 3.30)
Wavelength (Å)	1.71081	1.604970	1.282290	1.614380
R merge^a^ (%)	19.2 (124.8)	7.2 (77.8)	5.7 (72.5)	8.7 (122.9)
R_pim_^b^ (%)	4 (42.2)	2.4 (25.5)	2.3 (24.8)	2.9 (40.3)
CC½^c^	99.8 (48.1)	99.9 (87.7)	99.9 (88.4)	99.9 (69.4)
I/σ(I)	14.6 (1.9)	21.1 (3.2)	23.8 (3.3)	18.5 (2.1)
Completeness (%)	96.0 (77.4)	99.7 (99.1)	99.8 (99.7)	99.8 (99.4)
Anomalous multiplicity	11.5 (4.6)	5.3 (5.2)	5.3 (5.3)	5.3 (5.2)
Unique Reflections	39432 (3500)	17047 (3429)	18701 (4389)	18113 (3664)
FOM	0.73			
Refinement				
R work^d^ (%)	17.8			
R-free^d^ (%)	21.8			
Number of atoms:	8118			
Protein	8098			
Water	226			
Heteroatoms	20			
Ramachandran plot:				
Most favoured region	962 (96.2%)			
Allowed region	34 (3.4%)			
Outliers	4 (0.4%)			

Values in parenthesis are for the highest resolution shell.

^a^R merge = 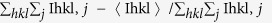 where I_hkl, j_ is the j^th^ intensity measurement of reflection hkl and < I > is the average intensity from multiple observations.

^b^Rpim =  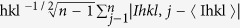/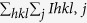 where n represents the multiplicity of the measurements.

^c^CC_½_ = Correlation coefficient between random half datasets^52^,^61^.

^d^R work = Σ_hkl_||Fo|-|Fc||/Σ_hkl_|Fo| for all data except 5% which were used for Rfree calculation.

**Table 2:** Height (σ) of the anomalous peaks determined by ANODE at Zn^2+^-edge, Co^2+^-edge and 40 ev below Co^2+^-edge for monomers A, B and C in ASU respectively.

Absorption edge	M1 site	M2 site	M3 site
Zn K-edge	/, /, 13 σ	21 σ, 15.4 σ, 14.2 σ	/, /, /
Co K-edge	13.5 σ, 12.2 σ, 10.4 σ	/, /, /	9.4 σ, 5.9 σ, 5.5 σ
Pre- Co-edge	/, 5 σ, /	4 σ, 5 σ, 4 σ	/, /, /

**Table 3:** PfTET3 activity at different metal-temperature using 5 mM Lys-pNa.

Metal	% Relative activity T = 20 °C	% Relative activity T = 50 °C	% Relative activity T = 85 °C
none	100	100	100
Zn^2+^ 0.1mM	55.5	61.6	52.3
Co^2+^ 0.1mM	50.3	67.0	229.4
Mn^2+^ 0.1mM	82.8	75.1	38.0
Zn^2+^ 1mM	13.5	15.5	35.4
Co^2+^ 1mM	14.3	35.6	107.2
Mn^2+^ 1mM	47.5	27.2	15.9

**Table 4:** Temperature dependence of PfTET3 kinetic parameters.

	Km (mM)	kcat (s^−1^)	kcat/Km (s^−1^ M^−1^)
PfTET3_Co_ 85 °C	0.62 ± 0.07	2436 ± 191	3.9*10^6^
PfTET3_Zn_ 85 °C	7.0 ± 1.41	2890 ± 412	4.1*10^5^
PfTET3_Zn_ 67.5 °C	8.7 ± 0.6	3500 ± 183	4.0*10^5^
PfTET3_Zn_ 50 °C	4.0 ± 0.6	959 ± 101	2.4*10^5^
PfTET3_Zn_ 35 °C	5.0 ± 0.8	707 ± 81	1.4*10^5^
PfTET3_Zn_ 20 °C	4.0 ± 0.9	45.7 ± 0.37	1.1*10^4^

**Table 5:** Activation parameters for PfTET3_Zn_.

	ΔH^‡^ (kJ/mol)	TΔS^‡^ (kJ/mol)	Log (A_obs_) (s^−1^)	ΔG^‡^(kJ/mol)
Range 20 °C–35 °C	116	53	10^22^	59.7
Range 35 °C–85 °C	23	−41.5	10^7^	64.5

**Table 6:** Glu207 pKa values of PfTET3_Zn_ and PfTET3_Co_ at 20 °C and 85 °C.

	20 °C	85 °C
pKa PfTET3_Zn_	5.9	4.75
pKa PfTET3_Co_	5.6	4.45
